# Regulation of medical devices outside the European Union

**DOI:** 10.1258/jrsm.2012.120037

**Published:** 2012-04

**Authors:** Susan Lamph

**Affiliations:** Guildford Medical Device Evaluation Centre (GMEC), The Royal Surrey County Hospital, Guildford, Surrey GU2 7XX, UK

## Introduction

The regulation of medical devices across the world is very varied, ranging from comprehensive to none. Over the past two decades, the number, range, and complexity of medical devices has increased. Regulation of these devices has also evolved due to an increasing awareness of the need for a more consistent approach to regulatory documentation. This will aid both manufacturers selling a product in more than one country, and the countries introducing regulation. Current initiatives are working towards manufacturers being able to produce a single set of documents that will fulfil the requirements of all regulatory authorities.

In 2001 the World Health Organization (WHO) published ‘*A model regulatory programme for medical devices: An international guide*’^1^ which provided a framework to assist member states in establishing regulatory programmes for medical devices. The guide was based on experiences from areas that had already established comprehensive regulatory programmes. The aim was to provide information to nations without medical device regulatory systems that would enable the production of internationally compatible regulations.

In 2003 the WHO published *‘Medical device regulations. Global overview and guiding principles’*,^2^ in which it highlighted the complexity of the medical device industry and identified issues related to regulation. This document provided guidance to member states wishing to create or modify their regulatory systems for medical devices.

The WHO is continually prompting harmonized medical device regulation through a range of initiatives, the most recent being the First Global Forum on Medical Devices held in Bangkok during September 2010. At the Bangkok meeting it was reported that: approximately 30% of countries have a developed framework for regulation of medical devices; approximately 30% of countries only have partial regulation of medical devices; remaining countries are either developing a framework or do not yet have any regulation.^3^

The Global Harmonization Task Force (GHTF) was founded in 1992 in response to a growing need for international harmonization of medical device regulation. This is a voluntary group comprised originally of representatives from the medical device regulatory authorities of the five founding members USA, European Union (EU), Japan, Australia and Canada. In 2006, membership was expanded to include the AHWP, International Organization for standardization (ISO), and the International Electrotechnical Commission (IEC).

The International Organization for Standardization (ISO) was set-up in 1946 to facilitate the international coordination and unification of industrial standards. ISO is a network of national standards institutes in 163 countries, and is now the world's largest developer and publisher of voluntary international standards.

ISO standards are widely adopted at regional and national level, and underpin the procedures and practices of medical device development, manufacture, quality control and conformity assessment requirements. In the global market these standards provide governments with a technical base for health, safety and environmental requirements, and aid the transfer of good practice and knowledge to developing countries.

Standards can be obtained from the ISO website (http://www.iso.org/iso/home.html). There are a large number of standards that relate to medical devices and some of the most important include: ISO 13485 *Medical devices – Quality management systems – Requirements for regulatory processes* (this is a specific medical device standard based on ISO 9001); ISO 10993 *Biological evaluation of medical devices*; ISO 14155 *Clinical investigation of medical devices for human subjects*; ISO 14971 *Medical devices – Application of risk management to medical devices*.

## Medical device nomenclature

Consistency and harmonization of nomenclature used for identification of medical devices is essential if information about the devices is to be exchanged between regulatory authorities. Two systems are recognized, the Universal Medical Device Nomenclature System (UMDNS) and Global Medical Device Nomenclature (GMDN) codes.

UMDNS terms are harmonized with the classification system used in the USA and are maintained by the Emergency Care Research Institute (ECRI). The system uses a unique 5-digit code to describe particular devices and exists in ten languages. The Asian Medical Device Nomenclature System (AMDNS) used in a limited number of Asian countries has been derived from the UMDNS and is fully compatible and interchangeable.

GMDN code is built according to EN ISO 15225 as a result of collaboration between the EU, European Free Trade Association (EFTA), USA and Canada. The GMDN database is a collection of terms that use unique 5-digit code to describe particular devices. The GMDN terms only exist in English but can be translated with special software. The database is maintained by the GMDN agency (a not for profit company based in the UK).

As a result of the work on regulatory harmonization, the regulation of medical devices continues to evolve. More countries and economic areas are adopting regulations; whilst those with established regulation are reviewing their requirements and updating existing regulation in line with the harmonization guidelines produced.

There is no medical device regulatory template that fits all. The responsibilities of regulatory authorities are broad, varied, and will depend on whether the country is an importer and/or exporter of medical devices. The three main stages of regulatory control are: pre-market, to ensure that the product to be sold meets standards of safety and performance; on-market, to ensure that the product is accurately labelled and advertised; post-market, to ensure the continued safety and effectiveness of the product in use.

## Classification of medical devices

The GHTF documents state that “regulatory controls should be proportional to the level of risk associated with a medical device.”^4,5^ To enable assessment of the level of risk, and therefore apply the correct regulations, medical devices are divided into different classes (Table 1). Some regulatory authorities have different risk classification systems for different groups of devices, for example general medical devices, Active Implantable Medical Devices (AIMD) and *In Vitro* Diagnostic (IVD) devices. Guidance on the risk-based method of classification for medical devices can be found in the GHTF documents.

**Table 1:** GHTF-proposed general classification system

Class	Risk level	General device examples	IVD device examples
A	Low	Surgical retractors/tongue depressors	Clinical chemistry analyser/prepared selective culture media
B	Low-moderate	Hypodermic needles/suction equipment	Vitamin B12, Pregnancy self-testing, Anti-Nuclear antibody, urine test strips
C	Moderate-high	Lung ventilator/bone fixation plate	Blood glucose self-testing HLA typing, PSA screening, Rubella
D	High	Heart valves/implantable defibrillator	HIV blood donor screening, HIV blood diagnostic

The pre-market requirements for medical devices vary for different regulatory bodies. A conformity assessment procedure, often dependant on the classification of the device will be required for all devices. Low-risk products may only require a supplier's declaration of conformity (SDOC), where the manufacturer is responsible for ensuring that the product complies with the relevant requirement and then produces a written self declaration statement. Higher-risk products will require a conformity assessment of the manufacturer's documentation, either by the regulatory authority or, as in Europe, an independent notified body.

The WHO has encouraged the use of mutual recognition agreements as a way of reducing trade barriers and harmonizing and increasing the pre-market regulation of medical devices. Mutual recognition is a process by which two or more countries agree to recognize some aspect of the others regulatory regime as being interchangeable with their own. This system enables a government in one country to approve products for marketing that are compliant with regulations in another. The European Union (EU) has a number of mutual recognition agreements with other regulatory organizations in various areas of medical devices regulation (Table 2).

**Table 2:** Countries with EU conformity recognition agreements

Country	Area of regulation covered	Exclusions
Australia	Conformity assessment	CE marked products manufactured outside the EU, Class 4 IVD devices
Canada		Not in operation for medical devices
Japan	Good laboratory practice	
New Zealand	Conformity assessment	CE marked products manufactured outside the EU
Switzerland	Conformity assessment	
Turkey	Conformity assessment	
USA	Quality system evaluations Product evaluations Post market vigilance reports	A modular agreement with some areas excluded

As part of post-market vigilance both manufacturers and regulatory bodies are expected to establish post-market surveillance and adverse events reporting procedures. Regulatory bodies should establish a national coordinating agency to receive and manage reports on issues related to medical devices. GHTF documents state that competent authorities should involve manufacturers in investigations of incidents and resolution of issues before notifying other national competent authorities.^6^

Manufacturer post-market surveillance of medical devices (or post-production monitoring as described in ISO 14971) should include: determination of changes made to the original medical device that effect risk assessment; a systematic process to evaluate product (not just customer complaints); inclusion of objective evidence in the risk management file; evaluation of any new hazards; determining whether there have been changes in the acceptability of risks as originally defined; inclusion of feedback and revisions of risk assessment/management as required.

Manufacturers are required to have proof in the form of documentation and an audit trail demonstrating that post-market surveillance is being performed. They also need to show that the data is being fed into appropriate national and international medical device monitoring system databases.

Australia, Canada, EU, Japan, and USA are the five founding GHTF member states. Regulations for medical devices in these markets are well established and regularly reviewed and updated. The regulations adopted in these areas are often used as guides for regulations being introduced in other countries. In many cases, approval for a medical device by one of these regulatory bodies is the main requirement for registration in countries that have limited resources and are only just introducing their own regulatory procedures.

Information on the regulation applied in the EU can be found in a separate NICE briefing document *CE marking process.*^7^

## USA

The Food and Drug Administration (FDA, www.fda.gov) regulates food, drugs, medical devices, biologics, cosmetics and radiation emitting products in the USA. Regulation of medical devices is overseen by the Centre for Devices and Radiological Health (CDRH). Medical devices are regulated under the Federal Food Drug and Cosmetic Act which originally came into force in 1938 and has since been regularly reviewed and updated.

Before any medical device can be marketed in the USA, a marketing application must be submitted to the FDA and clearance obtained. Currently the documentation submitted for registration of a medical device does not follow the requirements of a summary technical document (STED). USA and Japan are reviewing STED submissions to assess compliance with local requirements.^8^

Manufacturers and distributors of medical devices intended for sale in the USA are required to register annually with the FDA. This process is known as establishment registration. Information must be submitted electronically and includes details of the manufacturing and distribution site alongside a list of the medical devices marketed. The devices are added to the FDA medical device listing resister. This is the first step in the process to obtain FDA clearance to market.

The USA uses a risk-based classification for medical devices which takes into account the intended use of the product. The FDA has established a classifications database where generic types of devices are grouped into 16 panels. Each panel is then subdivided with devices assigned to one of the three classification classes (Table 3). The manufacturer determines the correct classification and details of the appropriate regulatory requirements for their product by checking the FDA website to ascertain which panel and subgroup applies. If the device does not appear to fit any of the panels, then clarification and advice on device classification will need to be obtained from the FDA.

**Table 3:** Classification of medical devices in the USA

Classification	Risk	Device type	Regulatory requirements
Class I	Low (present minimal potential for harm to the user).	Tongue depressors, arm sling, hand held surgical instrument examination gloves.	General controls. Not exempt – 510(k).
Class II	Low to medium (general controls alone are insufficient to assure safety and effectiveness).	X-ray systems, gas analysers, infusion pumps, surgical needles.	General controls with special controls. Not exempt – 510(k).
Class III	High (insufficient information exists to assure safety and effectiveness solely through general or special controls).	Replacement heart valves, silicon gel breast implants, pacemakers.	General controls with premarket approval (PMA).

Some class I/II devices are exempt from any more than general regulatory requirements, and do not require premarket notification 510(k). Details of exemptions are found on the FDA website.

The FDA requires manufacturers of regulated medical devices to follow the quality systems requirement known as current good manufacturing practices (CGMPs). This quality management system has, since its inception, undergone reviews and revision, and is now in line with the requirements of ISO 13485. The quality system regulations include requirements related to all aspects of device design, manufacturing, labelling, control, packaging and servicing. The FDA inspects manufacturing facilities to ensure quality system compliance. The FDA has determined that certain types of medical device are exempt from full GMP requirements. Details of exemptions can be found in the device classification database, but general control will still apply.

All medical devices, whatever class, require general controls to obtain FDA clearance to market. General controls state that devices must be manufactured under a quality assurance programme, be suitable for their intended use, be adequately packaged and properly labelled, and have FDA establishment registration and device listing. In addition, post-market surveillance and record keeping systems must be in place. Some products require special controls, these are device specific. Details of any special regulatory requirements will be found on the FDA classification database. Examples of special controls include specific labelling requirements and compliance with specific mandatory performance standards.

A 510(k) is a premarket submission made to the FDA to demonstrate that the device to be marketed is safe and effective. It must demonstrate that the device is substantially equivalent to a legally marketed device that is not subject to PMA. Documentation required with the 510(k) submission includes: description of the device; labelling information including draft promotional material; identification of predicate devices with narrative and tabular comparisons, and intended use indications; technical characteristics and principles of operation; software documentation; biocompatibility information; sterility information; statement or declarations of conformance to applicable standards and guidance documents; summaries of any performance testing.

The FDA review most 510(k) applications within 90 days. The device can be marketed once authorization from the FDA has been received. Authorization confirms substantial equivalence, clearance letters are posted monthly on the FDA website.

Premarket approval (PMA), a process of scientific and regulatory review to evaluate the safety and effectiveness, is required for all class III devices. This is the most rigorous of the regulatory procedures and is undertaken by the CDRH, who review PMA applications within 180 days. Guidance on the PMA process can be found on the FDA website.^8^ The information provided for PMA submitted to the FDA by the manufacturer must include: device description; description of the principles of operation of the device (including components) and properties relevant to clinical function; reports of key nonclinical studies; software documentation; sterility information; clinical studies; statistical analyses; published and unpublished literature including reports concerning the devices safety and effectiveness; basic labelling elements (statement of indication for use, contraindications, warnings, precautions, and instructions for use); environmental assessment. If approval is granted then the FDA issues a PMA approval letter, these are posted on the FDA website quarterly.

Investigational device exemption (IDE) allows devices to be used in clinical studies in order to collect data on safety and effectiveness to support a PMA application. Before the study can begin, the FDA issue a permit for use of the device. Clinical evaluation of a medical device not cleared for marketing requires: IDE approval by the FDA or an approved institutional review board; informed consent from all patients; labelling stating for investigational use only; study monitoring with results records and reports.

The Federal Food Drug and Cosmetic Act also allows for IVDs that are at different stages of development to be labelled for research use only (RUO) or investigational use only (IUO). This enables manufactures to evaluate limited-scale performance and potential clinical or informational usefulness of the test.^9^ Some of these diagnostic devices are exempt from IDE requirements; however these cannot be used for human clinical diagnosis unless the diagnosis is being confirmed by another medically established diagnostic product or procedure.

Once a device is on the market, manufacturers and distributors must follow post-market regulatory requirements. The FDA medical device reporting regulations require that manufacturers and users notify them of any malfunction, serious injury or death associated with a medical device. The Safe Medical Devices Act provides the FDA with two additional post-marketing activities; post-market surveillance for monitoring devices that have clearance to market and device tracking to maintain traceability of devices to the user. Information on adverse events involving medical devices can be found on the FDA website Manufacturer and User facility Devices Experience database (MAUDE).

USA is a member of the Asia-Pacific Economic Cooperation (APEC), which is involved in harmonization of medical device regulations between member states. USA is also a member of NAFTA, which promotes trade between Canada, USA and Mexico. USA has memorandums of understanding with Australia, Canada, Japan, and Switzerland relating to GMP and Good Laboratory Practice (GLP), and with China regarding safety of drugs and medical devices. Confidentially agreements related to the sharing of information on medical devices operate with Australia, Canada, Mexico, EU, Japan, New Zealand, Singapore and Switzerland.

## Russia

The registration and regulation of medical devices in Russia is complex and involves a number of different regulatory authorities. Roszdravnadzor (Federal Service on Surveillance in Healthcare and Social Development, www.roszdravnadzor.ru) is the competent authority and is responsible for registration, clinical safety and efficiency of all medical devices. Gosstandart (Federal Agency for Technical Regulation and Metrology) makes sure that all medical devices meet established Russian standards. A GOST-R quality certificate is required before any medical device can be imported into Russia. Rospotrebnadoz (Federal Service for Supervision in the Area of Consumer Rights and Welfare Protection) is responsible for ensuring that all medical devices that come into contact with the human body, or which may otherwise negatively affect patients or doctors, meet sanitary and epidemiological regulations. A Sanitary-Epidemiology Conclusion (Hygiene Certificate) is required before any medical device can be imported into Russia. Russia uses a risk based classification system for medical devices with four classes (1, 2a, 2b, and 3) where class 1 is the lowest and class 3 the highest level of risk. Roszdravnadzor reviews applications for registration. Roszdravnadzor, as part of the review process, defines which aspects required for performance testing must be carried out in Russia. Medical devices need a GOST-R quality certificate and Hygiene Certificate before they can be imported for performance testing. Roszdravnadzor is responsible for post-market surveillance. Russia is a member of APEC which is involved in harmonization of medical device regulations between member states.

## Asia

### Asian Harmonisation Working Party (AHWP)

AHWP is a non-profit making organization that is working in association with member countries and the GHTF to harmonize medical device regulation in Asia. The current membership includes Abu Dhabi (UEA), Brunei Darussalam, Cambodia, Chile, China, Chinese Taipei (Taiwan), Hong Kong SAR, India, Indonesia, Jordan, Saudi Arabia, Korea, Laos, Malaysia, Myanmar, Pakistan, Philippines, Singapore, South Africa, Thailand, and Vietnam.

AHWP is currently working on producing a Safety Alert Dissemination System (SADS) based on experience in USA, EU, Australia and Japan. The aim is to have a single data system that can be shared by member nations.

### Association of Southeast Asian Nations (ASEAN)

ASEAN was established in 1967 with the signing of the Bangkok declaration by Indonesia, Malaysia, Philippines, Singapore and Thailand. Since then Brunei Darussalam, Vietnam, Laos, Myanmar and Cambodia have joined to make up what is today a ten member organization. In 2007 the ASEAN leaders signed the Cebu Declaration on the Acceleration of the Establishment of an ASEAN Community by 2015.

The aim and purpose of the organization has been to accelerate economic growth, social progress and cultural development across the region. Alongside this, they aim to promote active collaboration and mutual assistance in matters of common interest in economic, social, cultural, technical, scientific and administrative fields.

The Medical Device Product Working Group (MDPWG) is responsible for medical device regulatory harmonization within ASEAN. They are working to establish a unified set of rules based on GHTF guidelines which will institute a common format for medical device application dossiers. The ASEAN Medical Device Directive (AMDD) identify the basic requirements for medical device safety and performance, include a classification system, a Common Submission Dossier Template (CSDT) and ASEAN-wide post marketing alert system. The AMDD will not be a law in ASEAN countries, but all member countries will be required to pass laws with the same provisions.

### Asia-Pacific Economic Cooperation (APEC)

APEC was established in 1989 by Australia, Brunei Darussalam, Canada, Indonesia, Japan, Korea, Malaysia, New Zealand, the Philippines, Singapore, Thailand and USA. Since then China, Hong Kong, Chinese Taipei (Taiwan), Mexico, Papua New Guinea, Chile, Peru, Russia and Vietnam have joined taking the membership to 21.

APEC held a series of seminars in 2008 and 2009 on harmonization of medical device regulations. The aim of these seminars was to help members of APEC economies develop robust regulatory systems for medical devices. Training programmes and workshops coordinated with the GHFT have since been arranged, aimed at strengthening and harmonizing regulatory requirements within the economic area. The APEC Harmonization Centre (AHC) was launched in 2009 to continue the work towards harmonizing regional regulatory priorities and practices for medical devices.

## China

China is a member of AHWP and APEC. State Food and Drug Administration (SFDA, http://eng.sfda.gov.cn), part of the Ministry of Health, is responsible for the regulation of both internally manufactured and imported medical devices. The SFDA regulations follow the USA FDA model. The first regulations for the supervision and administration of medical devices came into effect in April 2000. This was followed in August 2004 with provisions governing the registration of medical devices. In April 2007, new IVD regulations were introduced which detailed specific requirements for IVDs.

Medical devices are classified into three classes I, II and III according to risk, with class I being the lowest and class III the highest level of risk. The regulations are complex and require manufacturers to comply with a number of local and international standards.

In addition to SFDA approval, seven categories of medical device require China Compulsory Certification (CCC). These include medical X-ray equipment, haemodialysis equipment, hollow-fibre dialysers, extra-corporeal blood circuits for blood purification equipment, electrocardiographs, implantable cardiac pacemakers, and artificial heart-lung machines. The administration of the CCC mark is handled by the China Quality Certification Centre.

Products produced in China can carry the ‘China Export’ symbol, which is not a registered trademark. This symbol is very similar to the CE mark applied to products that are compliant with EU standards, so care should be taken not to confuse the two symbols.

Post -market surveillance of medical devices in China is still in development. Surveillance for medical devices is currently limited to adverse event reporting and recall, alongside limited inspection of manufacturers by the SFDA. A national database for the collection of adverse event reports and surveillance inspection reports is not yet established.

## Hong Kong

Hong Kong is a member of AHWP and APEC. The Department of Health, The Government of the Hong Kong Special Administrative Region is responsible for the regulation of medical devices. The first stage of the Medical Device Administrative Control System (MDACS) began in November 2004 and is still ongoing with the latest stage being introduced in December 2009. Guidance documents were published in January 2010. A full regulatory programme has not yet been introduced.

Hong Kong has adopted a risk based classification system based on the GHTF guidance. Four classes of devices I, II, III and IV are identified where class I is the lowest and IV the highest level of risk. Conformity assessment requirements follow those outlined in GHTF guidance documents and include the need for a STED, quality management system and provision for manufacturer post market surveillance.

The MDACS has a medical devices adverse incident reporting system for both manufacturers and users. Reporting forms are available on the MDACS section of the Ministry of Health website alongside recalls and alerts.

## India

India is a member of AHWP. The regulation of medical devices in India is currently managed by the Central Drug Standards Control Organization (CDSCO) which is part of the Ministry of Health and Family Welfare. Medical devices are defined as drugs and regulated under the Drug and Cosmetics Act 1940 and the Drugs and Cosmetic Rules 1945. However, there are proposals for a new regulatory authority to be formed when new medical device regulations which are in the pipeline become law.

Under the current system, only imported implantable devices, diagnostic kits and sterile devices require registration. The devices are classified as either life-saving medical equipment or non life-saving medical equipment. Medical devices not defined as drugs only require an import licence, quality systems do not exist.

The new regulations based on GHTF recommendations will include all medical devices. A requirement for a quality system management and a risk based classification system will be included. It is proposed that for Class A devices, the lowest risk level, manufacturers will perform their own conformity assessment. Class B, C and D devices will require assessment by an authorized notified body. Imported medical devices that have CE Mark, FDA approval or equivalent will be allowed on the market without undergoing separate conformity assessment procedures. The documentation for registration will follow the GHTF STED guidelines.

Post-market surveillance is undertaken by the Central Licensing Approval Authority (CLAA) at the CDSCO who collect data on post-market events from manufacturers. The new regulations will expand post-market reporting requirements.

## Middle East

### Iran

The Iran Medical Equipment Department within the Ministry of Health and Medical Education is responsible for the regulation of medical devices. The Medical Equipment by-law part of the Ministry of Health, Medical cure and Medical Education Act contains the regulatory requirement.

Medical devices are classified according to the GHTF guidelines into four classes A, B, C and D, where class A is the lowest and D is the highest level of risk. This classification structure generally corresponds to that used in the EU. Documentation required for registration is similar to that required for FDA approval or EU CE marking and follows the format of the GHTF STED.

A post-market surveillance system is managed by the General Department for Medical Equipment.

### Israel

The Ministry of Health is responsible for the regulation of medical devices in Israel. All companies wishing to import medical equipment or devices must be registered with the Ministry of Health and have a local agent or distributor. Importers are required to provide certification issued by a competent authority to show that the medical device has obtained USA FDA approval, EU CE marking, Australian, Canadian or Japanese regulatory approval. In addition, all products will require labelling and instructions in Hebrew.

### Jordan

Jordan is a member of AHWP. The Jordan Food and Drug Administration, a department within the Ministry of Health, oversees the laws and regulations related to medical devices in both the public and private health sector. There is no information on the regulation or post-market surveillance of medical devices in English on the Hashemite Kingdom of Jordan Ministry of Health website.

Medical devices that have obtained USA FDA approval, EU CE marking or Japanese regulatory approval and have been certified for use in their country of origin do not require regulatory review prior to registration for use in Jordan.

### Saudi Arabia

The Saudi Food and Drug Authority (SFDA) was established in 2003 and is an independent authority that reports to the council of ministers. SFDA is responsible for the regulation of medical devices in Saudi Arabia. Currently, interim medical device regulations adopted in 2008 are in place and will apply until the *medical devices comprehensive law* is approved. The regulations ensure that only medical devices that have been authorized by one of the founding GHTF members (Australia, Canada, EU, Japan and USA) have access to the Saudi Arabian market.

A Medical Device National Registry (MDNR) has been launched by the SFDA. This will profile the industry and provide a data base of all establishments, manufacturers, agents and suppliers working in the medical device field in Saudi Arabia. Alongside this, a licensing and surveillance system has been established. Saudi Arabia is a member of AHWP and has signed up to the SADS for post-market surveillance of medical devices.

### United Arab Emirates

UAE is a member of AHWP. The Drug Control Department within the Ministry of Health is responsible for supervision and direction of the regulation of medical devices in the UAE. The UAE has adopted regulations based on the GHTF guidelines and existing EU, Australian TGA and USA FDA regulation, alongside the UAE Pharmacy Law No4 for 1983. A risk based classification system with four classes I, IIa and b, III and IV has been adopted where class I has the lowest and class IV the highest level of risk.

The manufacturer or local authorized representative is required to apply to the Technical Section of the Drug Control department to register all devices before they can enter the market. The documentation required is based on the GHFT STED model. All manufacturers require ISO 13485 certification to conform to quality management requirements. Medical devices that have approval from recognized regulatory authority (EU, USA, Australia, Canada or Japan) can use an abridged process for registration.

The UAE has an established post-market surveillance and vigilance system which requires all manufacturers to maintain distribution, complaint and adverse event records.

## Africa

In 2005, the WHO reported that only 7% of the 46 sub-Saharan African countries had National Medicines Regulatory Authorities (NMRA) in place. Of the remaining countries, 63% had minimal regulation and 30% had no regulation.^10^ A number of international organizations including the African Organization for Standardization (ARSO), The African Network for Drugs and Diagnostic Innovation (ANDI), African Union (AU) and United Nations Economic Commission for Africa (UNECA) have been established to promote harmonization of procedures and standards within the African continent.

## Caribbean, Central and South America

The Pan American Health Organization (PAHO) is a WHO group that includes representatives from the majority of the countries in the Caribbean, Central and South American area. One of the aims of the organization is to improve the safety and technical efficacy of medical devices within the region. PAHO has designated ECRI to work with them to establish and coordinate a medical devices safety programme for the area (2007–11). ECRI have established a centralized database for reporting of adverse incidents and near misses associated with medical devices. ECRI analyse reports and make recommendations on safe practice to prevent subsequent problems.

### South America

Mercado Común del Sur (MERCOSÚR), the common market of the south, was set up in 1991 by Argentina, Brazil, Paraguay and Uruguay under the Treaty of Asuncion. The 1994 Treaty of Ouro Preto gave the organization a wider international status and formalized a customs union. The membership of Mercosur has now expanded and includes Venezuela and five associate members: Chile, Bolivia, Colombia, Ecuador and Peru. Negotiations for an inter-regional association agreement between Mercosur and the EU began in 1999 but were later suspended. However in May 2010, talks resumed with the theme of the summit ‘towards a new stage in the bi-regional partnership: innovation and technology for sustainable development and social inclusion.’ Harmonization of the regulation for medical devices within the Mercado area is one of the aims of the organization. Currently the group is working towards each country having the same regulatory requirements and a single quality management system. Medical devices still require approval in each member state, but the eventual aim is to have a single approval scheme similar to that of the EU.

For products that may already have FDA or EU (CE mark) approval, an independent approval process has been established in South America. A Free Sale Certificate (FSC) or a Certificate to Foreign Government (CFG) obtained from the country of origin confirms that a product is approved for sale there. These certificates include a product description with specific product numbers and identify the manufacturing site. They can enable medical devices to be exported to South America without restriction.

### Mexico

Federal Commission for the Protection against Sanitary Risk (COFEPRIS), a division within the Mexican Secretaria de Salud, is responsible for the regulation of medical devices and IVDs. All manufacturers must have an office or distributor in Mexico who is responsible for the registration of the medical device with the Secretaria de Salud before it can be imported into Mexico.

Classification of medical devices is according to a COFEPRIS catalogue for instrumental and medical equipment. The catalogue is divided into three areas: instrumental, medical equipment and materials for prosthesis and orthesis. IVDs are dealt with separately. This is a risk based system with three classes I, II and III. The documentation required for registration and review process is dependent on the class of the medical device.

No local GMP standards exist. A quality management system certificate ISO 13485 or official proof of compliance with another national quality system is required for all imported devices. Post-market controls are managed by COFEPRIS and include adverse event reporting, tracking and recall.

Mexico is a member of APEC which is involved in harmonization of medical device regulations between member states. Mexico is also a member of NAFTA which promotes trade between Mexico, USA and Canada.

### Cuba

Centro de Control Estatal de Equipos Médicos (CCEEM) was established in 1992 is part of the Cuban Ministry of Public Health and is responsible for the registration and control of medical devices in Cuba. Cuba has a comprehensive and advanced regulatory programme for medical devices which is based on GHTF recommendations and includes a risk based classification system. The regulations encompass recommendations and conditions outlined in GHTF documentation for: role of standards and assessment of medical devices; quality requirements; post- market surveillance; essential principles; labelling for medical devices.

## DECLARATIONS

### Competing interests

None declared

### Funding

NICE

### Ethical approval

Not applicable

### Guarantor

Chris Pomfrett

### Contributorship

Susan Lamph wrote the first version of the manuscript while working for GMEC, an independent academic External Assessment Centre working under contract to NICE.

Dr Chris Pomfrett does not claim authorship, but acted as supervising editor for this work in NICE. Chris Pomfrett is a Technical Adviser working in the Medical Technologies Evaluation Programme (MTEP) at NICE

### Acknowledgments

None
